# The Yekaterinburg headache initiative: an interventional project, within the Global Campaign against Headache, to reduce the burden of headache in Russia

**DOI:** 10.1186/1129-2377-14-101

**Published:** 2013-12-24

**Authors:** Elena R Lebedeva, Jes Olesen, Vera V Osipova, Larisa I Volkova, Guzyal R Tabeeva, Timothy J Steiner

**Affiliations:** 1 Department of Neurology, Urals State Medical University, Yekaterinburg, Russian Federation; 2 Department of Neurology, University of Copenhagen, Copenhagen, Denmark; 3 Department of Neurology, First Sechenov Moscow State Medical University, Moscow, Russian Federation; 4 Department of Neuroscience, Norwegian University of Science and Technology, Trondheim, Norway

**Keywords:** Action research, Burden of headache, Health care, Service delivery and organization, Russia, Global campaign against headache

## Abstract

**Background:**

As major causes of global public ill-health and disability, headache disorders are paradoxically ignored in health policy and in planning, resourcing and implementing health services. This is true worldwide. Russia, where the prevalence of headache disorders and levels of attributed disability are well in excess of the global and European averages, is no exception, while arcane diagnoses and treatment preferences are an aggravating factor. Urgent remedial action, with political support, is called for.

**Methods:**

Yekaterinburg, in Sverdlovsk Oblast, is the chosen centre for a demonstrational interventional project in Russia, undertaken within the Global Campaign against Headache. The initiative proposes three actions: 1) raise awareness of need for improvement; 2) design and implement a three-tier model (from primary care to a single highly specialized centre with academic affiliation) for efficient and equitable delivery of headache-related health care; 3) develop a range of educational initiatives aimed at primary-care physicians, non-specialist neurologists, pharmacists and the general public to support the second action.

**Results and conclusion:**

We set these proposals in a context of a health-care needs assessment, and as a model for all Russia. We present and discuss early progress of the initiative, justify the investment of resources required for implementation and call for the political support that full implementation requires. The more that the Yekaterinburg headache initiative can achieve, the more likely it is that this support will be forthcoming.

## Background

In Western Europe, where the burden of headache is best measured [1,2], the lives of about 17% of adults are affected by headache disorders to the extent that they would benefit from professional medical care [3]. Most of these people (about 14%) have migraine; some have frequent episodic tension-type headache (TTH). The various causes of headache on ≥15 days/month, including medication-overuse headache (MOH), together account for up to 3%.

The principal consequences of these disorders are high levels of population ill-health and disability and a heavy societal economic burden [1,2,4]. Migraine on its own is the seventh highest specific cause of disability worldwide [5,6]. In the European Union, the annual financial cost of headache, due mostly to lost productivity, has been estimated in one study at €44 billion [7] and in another at more than €100 billion [4].

These burdens are reducible, since most headache disorders can be treated effectively [8]. Moreover MOH, a very substantial contributor [4], is iatrogenic and could therefore be eliminated. Nevertheless, throughout the world, the health-care need that headache disorders generate is largely unmet [2,3].

### Headache disorders in Russia

Reliable data are available from a recent countrywide survey [9,10]. Of 2,025 adult participants, nearly two thirds (62.9%) reported headache in the previous year. The estimated 1-year prevalence of migraine was 20.8%, of TTH 30.8% and of headache on ≥15 days/month 10.4%. Of the last, a majority of cases (68.1%) were associated with overuse of acute headache medications. Table 1 puts these findings into the global context: the prevalence of migraine in Russia is nearly 50% higher than the world average, and of headache on ≥15 days/month over three times the world average.

**Table 1:** One-year prevalences of disabling headache disorders in adults in Russia, and world averages

**Headache disorder**	**Russia **[10]	**Global average**
All (*ie*, any headache in the last year)	63%	46% [1]
Migraine	21%	14.7% [5]
Headache on ≥15 days/month	10%	3% [1]

Much disability results: the estimated productivity losses to headache in Russia account for 1.75% of gross domestic product (GDP) [11]. Clearly these statistics signal very substantial and unmet health-care needs of people with headache in Russia. While the health-care needs of people with headache are to a large extent unmet in all countries of the world [2], in Russia there are particular reasons for this beyond the high prevalence of these disorders. The prevailing view among the majority of Russian primary-care physicians (PCPs), and many neurologists, is that headache signals an organic brain lesion [12,13]. In consequence, almost all patients who complain of headache are referred, mostly unnecessarily, for investigations and/or neurological consultations [14] – a practice reinforced by official “standards” [15,16]. This is obviously wasteful [2] but, worse, the non-specific and clinically unimportant changes often found on investigations are interpreted as evidence of organic brain lesions. Patients then receive erroneous and sometimes arcane diagnoses such as “dyscirculatory encephalopathy” or “autonomic dysfunction with headache paroxysms” [12,13]. These unhelpful practices are reinforced by the system of obligatory medical insurance, whereby such diagnoses provide not only the right to extended sickness benefit for the patient but also enhanced reimbursements to the treating institution. Furthermore, these erroneous diagnoses lead to treatment not with medications effective for migraine or TTH but with inappropriate vasoactive, nootropic or venotonic agents [14].

### Education failure lies behind health-care failure throughout the world

These practices arise, almost exclusively, from inadequate understanding of headache disorders [13]. According to a worldwide survey published by WHO, a key reason for failure of good health care for headache in all countries is that physicians receive little training in diagnosing and managing headache disorders [2], a reflection of the paradoxically low priority accorded to these common and burdensome disorders. PCPs in particular, lacking confidence when treating affected patients, over-investigate, offer suboptimal treatment and achieve poor and discouraging outcomes.

Action is urgently called for [11], taking note not only of the effective and cost-effective treatments available [8] but also of persuasive recommendations that headache services should have their basis in primary care. Commentary to WHO’s survey placed strong emphasis on the *need* for this [2], partly because of numbers – it is impossible for all or even a large proportion of patients with headache to be seen by specialists [3] – and partly because it is wasteful to send patients unnecessarily to specialist care. Most people seeking health care for headache require only the skills routinely available to all practising doctors coupled with a basic understanding of the common headache disorders [3]. But in WHO’s survey, need for better professional education ranked far above all other proposals for change [2].

### Sverdlovsk Oblast

In the Ural Federal District of Russia, Sverdlovsk Oblast has its administrative centre in the city of Yekaterinburg. Its population is 4.3 million (2010 Census), of whom >90% are ethnic Russians. It is a region of relatively high wealth, third in economic potential in the country after Moscow and St Petersburg.

The State health-care system of Sverdlovsk is free to users, although, except for certain chronic illnesses, patients must meet most drug costs in full. It is also based on sound infrastructure. Primary care is well-established: 1,260 PCPs work in primary health-care centres or in district-based polyclinics or hospitals throughout the region [information provided in 2012 by Sverdlovsk Ministry of Health and Social Development]. More advanced specialist services are provided in 234 regional centres, and 10 interregional municipal centres offer highly specialized services in disciplines including neurology. The Urals State Medical University (USMU) in Yekaterinburg is internationally recognized.

For these reasons, Yekaterinburg was the chosen centre for an interventional project in Russia within the Global Campaign against Headache [17,18] – the Yekaterinburg headache initiative (YHI). The Campaign is directed by *Lifting The Burden* (LTB), a UK-registered not-for-profit non-governmental organization in official relations with WHO [19]. The aim of the Campaign is to improve the management of people affected by headache everywhere in the world.

### Objectives

The immediate objective of YHI is to develop effective and cost-effective health care for headache disorders locally, based on existing health-service organization and infrastructure. The longer-term objective is to reduce the individual and societal burdens of headache by meeting the need for headache care throughout Sverdlovsk Oblast. Ultimately, it is intended to replicate the initiative in other States as a health-care model for headache for all of the Russian Federation.

## Methods

The programme consists of three actions, not necessarily proceeding in sequence.

### Action 1: to raise awareness of need for improvement

The evidence needed for an awareness campaign is already available [1,2,10], and entirely sufficient to support a chain of four arguments:

•the humanitarian burdens – pain, suffering and disability – of headache disorders are high [1,2,10];

•the consequential societal costs in productivity losses are very high [4]: in Russia, they account for 1.75% of GDP [11], which is over one third of all expenditure on health care [20];

•greater investment in health care for headache should be cost-saving overall because lost-productivity costs of headache hugely outweigh health-care costs [2,4];

•health-care resources currently allocated to headache are used wastefully [2,11-16], and could be more effectively redeployed, limiting the investment needed.

This evidence, and much more that is to come (see later), must be brought to the attention of government, whose awareness and recognition of the scale and scope of the problem are needed to secure investment in State health services.

### Action 2: to design and implement a model for efficient and equitable headache-related health care in Sverdlovsk Oblast

LTB, in collaboration with the European Headache Federation (EHF), has developed a three-tier model for headache services in Europe [3]. It is based in primary care, with referral channels upwards and educational support downwards. Within the model, about 90% of headache patients are managed in primary care (level 1); about 1% require specialist care (level 3); the intermediate 9-10%, whilst not requiring this, nonetheless present diagnostic or management difficulties that call for a higher level of expertise (level 2) than can be expected of the average PCP. Except in emergencies, entry is at level 1: a key feature of the model is that each lower level has a gatekeeper role to the level(s) above, which, when the model is fully in place, ensures both efficiency and equitable access.

The action required is to implement this model, adapting it as necessary and building it into the present health-care system of Sverdlovsk Oblast.

### Action 3: educational initiatives

These initiatives, undertaken hand-in-hand with and in support of action 2, must be aimed at PCPs at level 1, general (or non-headache specialist) neurologists at level 2, pharmacists and the general public (people with headache). Curricula for professional training should be endorsed by the Russian Headache Research Society (RHRS). Teaching should be given by specialists, with support from USMU and, ideally, from the Ministry of Health, and accredited for continuing medical education (CME) purposes.

The amount of knowledge required by PCPs at level 1 is not great in order to enable effective management of most people with headache, but it must be imparted to large numbers of PCPs. A 1-day (6-hour) course at this level should focus on the presentation, aetiology, diagnosis and management according to European guidelines [8] of the three most common and important headache disorders in public-health terms (migraine, TTH and MOH). Nonetheless, it should also include other headache disorders likely to be encountered in primary care, which need to be correctly recognised, and provide clear guidance on when to refer. The course should be repeated until all PCPs within the scheme have been reached.

A higher level of knowledge is required by neurologists at level 2. A 1-day (6-hour) advanced course to supplement the level-1 course should cover cluster headache, a range of other primary headache disorders that are more difficult to diagnose and/or manage, some secondary headaches and comorbid disorders.

Specialist knowledge for level 3 requires theoretical and practical training provided by national and international experts. The curriculum must cover all headache disorders.

High-street pharmacists are often the first (and sometimes only) source of information to the public about headache disorders and treatments for them. Their training should cover recognition and treatment of the common headache disorders, the dangers of medication overuse, and warning indications of serious headache. Evening courses of 2 hours are most likely to be taken up.

Public education through the media, organized by RHRS, should focus on the recognition of different headache types, their causation and steps that might be taken to prevent them, appropriate and inappropriate use of medication, and when to seek professional advice.

## Results

A meeting at the Sverdlovsk Ministry of Health in 2012 gained government acknowledgement of the public-health priority of headache disorders. It resulted in endorsement of the concept of structured, tiered headache services based in primary care (Table 2), and agreement to support the project and this service model. It has not resulted so far in actual support.

**Table 2:** Proposed three-level model for headache services in Sverdlovsk Oblast

**Level**	**Service**
1	First point of access to health care for everyone with headache, provided in the primary health-care centres or district-based polyclinics by trained primary-care physicians
2	Provided in all 10 interregional municipal centres and some of the 234 regional centres by neurologists with an interest and more advanced (but not specialist) training in headache
3	A single highly specialized centre in Yekaterinburg with academic affiliation

Implementation of the service model is necessarily step-wise, with empirical assessment of progress. A first step is the development of management aids, particularly for use by non-specialist physicians: diagnostic aids, management guidelines, disability and outcome measures and patient information leaflets. All of these have been produced by LTB for Europe [21,22], with multicultural relevance achieved through input from multiple countries (including Russia), and translated into Russian.

Of the headache services themselves, level 3 must be established first because it is needed to support, mainly through education but also clinically, both development and effective operation of levels 1 and 2. Headache disorders are a recognized specialty at USMU, but scarce resources hinder creation of a university-based level-3 centre. Pragmatically, it is necessary to follow the path that the present system permits, and build upon what is available. In the private sector, the Europe-Asia Headache Centre (EAHC) was established by EL in Yekaterinburg over two years ago, and USMU both recognises and collaborates with it clinically and academically. EAHC was modelled on the Danish Headache Centre (DHC), one of the leading centres in the world. EAHC has established a research programme, already supporting five PhD students. Using a Russian modification of the validated Danish electronic semistructured interview [23], EAHC has built a database of over 1,000 patients, which will expand over time as a valuable research resource.

Much of the educational action has been implemented. EL and LV have organized a series of half-day teaching courses. Fifty participants, after completing five such courses, will receive diplomas in headache medicine from USMU, establishing the expertise needed for level-2 headache care. To encourage CME, several scientific meetings exclusively on headache have been organized, and headache sessions have been included during pain and neurology congresses.

For the future of level 1, the undergraduate curriculum for medical students has been updated in headache – a major step in dealing with the lack of training in headache identified by WHO [2]. Each future student will for the time being receive 6 hours of teaching on headache disorders; this is not enough, but it is 50% more than the global average of 4 hours [2], and therefore a promising start.

## Discussion

For planning purposes, an estimate of service requirement at each level is available from a European needs-assessment [3] (Table 3). The 120 FTE PCPs at level 1 may in practice be, say, four times this number committing, on average, 25% of their time to headache; or the workload may be spread between all PCPs. However it is divided, this allocation represents almost 10% of total capacity in primary care. If this appears excessive, it should be recognized that headache is the most frequent single cause of consultation in primary care everywhere [24]. This estimate assumes that 90% of presenting patients are managed at level 1 [3], whereas in Sverdlovsk some workload may be transferred to level 2. This itself requires 21 FTEs (again it should be recognized that headache is the most frequent single cause of consultation in neurological practice [24]); as there are 234 regional centres, demand at level 2 can be met by one neurologist in each centre committing 10% of his or her time to headache, which suggests spare capacity for some transfer of workload from level 1. Assuming the effective operation of levels 1 and 2, level 3 requires two FTEs, or 4–5 specialists committing 40-50% of their time to headache. While all these estimates are based on European averages [3], it has been noted that headache disorders are considerably more prevalent in Russia than in Western Europe. This is especially true of disorders characterized by headache on ≥15 days/month, which place especially high demands on health services [4]. They may, therefore, be underestimates. This will be established empirically, but not unless and until the services are in place.

**Table 3:** **Service requirement in Sverdlovsk Oblast (estimates based on a European needs-assessment**[3]**)**

	**European standard **[3]	**Requirement in Sverdlovsk Oblast**
**Level 1**	One full-time equivalent (FTE) PCP for every 35,000 of the population	About 120 FTE PCPs
**Level 2**	One FTE physician per 200,000 people	21 FTE neurologists
**Level 3**	One FTE specialist per 2,000,000 people	2 FTE headache specialists

*FTE* full-time equivalent; *PCP* primary-care physician.

Step-wise implementation of this model is not without difficulties: it is built on interdependent levels, and all need to be in place and functioning for it to work efficiently and equitably as envisioned (in many countries, it is ineffective operation of levels 1 and 2 that leads to suffocation of headache services at level 3 [2]). Implementation in a country like Russia is top-down: level 3 is the driver, so the continued development and expansion of EAHC is of primary importance at this stage. Research at EAHC is already at high level and volume. Its teaching programme is well-developed and expanding: YHI is already delivering the training needed adequately to equip level 2 and, as this is completed, the focus can shift more towards level 1. It is lack of clinical referrals that poses the biggest impediment to its future success in a system of reimbursement that promotes over-investigation without ensuring correct diagnosis or management, while at the same time discouraging referral upwards through perverse incentives [13-16]. Education may improve skills and change behaviour, but the solution is ultimately political. While accredited training courses can be arranged for PCPs (and high-street pharmacists), these can achieve nothing unless these practitioners are willing to participate. Official “standards” [15,16] must also change.

### Next steps

YHI is a demonstration of what can be achieved with very limited resources and, so far, without direct government support (albeit with government endorsement). It is not yet a model for all Russia (one of our objectives): placement of level 3 in a private centre does not meet the European standard, which requires access to all specialties so that all secondary headaches can be managed [3]. USMU provides back-up for this purpose, but across the private/State-funded interface, raising issues of access. Equitable access, a *sine qua non* of the fully-implemented model, will be achieved when EAHC gains Ministry recognition as a level-3 centre, receiving reimbursements from public funds.

Both practical and financial government support, along with political commitment, are required now if there is to be further progress. In particular, levels 1 and 2 cannot be adequately established without Ministry drive because this action involves service organization, not only education. With compelling economic arguments behind us [1,2,4,10-16], we will press for this support.

## Conclusions

This ambitious initiative is coming to fruition. The development of the Europe-Asia Headache Centre in Yekaterinburg into a highly-specialized level-3 headache centre, with the full (non-financial) support of the Urals State Medical University, is far advanced. Considerable inroads have been made into the local teaching requirement, directed especially towards level 2. In the present universal climate of limited resources, nothing is achieved quickly, and local government support is essential to reach project fulfilment. The more that the Yekaterinburg headache initiative achieves meanwhile – and demonstrates to be effective – the more likely it is that this support will be forthcoming.

## Abbreviations

CME: Continuing medical education; EAHC: Europe-Asia Headache Centre; EHF: European Headache Federation; FTE: Full-time equivalent; GDP: Gross domestic product; LTB: Lifting The Burden; MOH: Medication-overuse headache; PCP: Primary-care physician; RHRS: Russian Headache Research Society; TTH: Tension-type headache; USMU: Urals State Medical University; WHO: World Health Organization; YHI: Yekaterinburg headache initiative.

## Competing interests

EL is Director of the Europe-Asia Headache Centre, and Representative of Russia in EFNS Scientific Panel on Headache. VO and GT are, respectively, General Secretary and President of the Russian Headache Research Society. TJS is a director and trustee of *Lifting The Burden*.

## Authors’ contributions

JO and EL instigated the project. TS produced the structured outline of the YHI as a project within the Global Campaign against Headache, which was refined by EL, JO and TS. EL founded the EAHC, developed it under guidance from JO and expanded it within the project with support from JO and LV. EL, LV and TS sought and obtained endorsement from the Ministry of Health. EL was primarily responsible for clinical implementation, with support from LV. EL, LV, VO, GT and JO developed and implemented the educational initiatives. TS drafted the manuscript. All authors reviewed the first and subsequent drafts, and approved the final version.
